# Dexmedetomidine sedation during the nighttime reduced the incidence of postoperative atrial fibrillation in cardiovascular surgery patients after tracheal extubation

**DOI:** 10.1186/s40560-015-0092-5

**Published:** 2015-05-30

**Authors:** Ayuka Narisawa, Masaki Nakane, Takako Kano, Nozomi Momose, Yu Onodera, Ryo Akimoto, Tadahiro Kobayashi, Masahiro Iwabuchi, Masayuki Okada, Yoshihide Miura, Kaneyuki Kawamae

**Affiliations:** Department of Anesthesiology, Yamagata University Faculty of Medicine, 2-2-2 Iida-nishi, Yamagata City, Yamagata 990-9585 Japan; Department of Intensive Care, Yamagata University Hospital, 2-2-2 Iida-nishi, Yamagata City, Yamagata 990-9585 Japan; Department of Dental Anesthesiology, Health Sciences University of Hokkaido, 1757 tobetsu-cho kanazawa, Ishikari-gun, Hokkaido 061-0293 Japan

**Keywords:** Dexmedetomidine, Atrial fibrillation, Cardiovascular surgery, After tracheal extubation, Intensive care unit

## Abstract

**Background:**

Dexmedetomidine (Dex) provides sedation and analgesia by acting on central alpha-2 receptors and is suitable for use after extubation because it has little respiratory depression. Considering the sympathoinhibitory and anxiolytic action of Dex, there is the possibility that Dex might reduce the incidence of atrial fibrillation (AF), which is recognized as a common complication after cardiovascular surgery. We investigated whether the postoperative incidence of AF decreased in patients who received Dex only during the nighttime in the intensive care unit (ICU).

**Methods:**

We retrospectively reviewed ICU charts to determine the incidence of AF and associated factors during the 2-day period after tracheal extubation in patients who underwent cardiovascular surgery from November 2009 to November 2010. The patients were divided into a Dex group (*n* = 16) and a non-Dex group (*n* = 29).

**Results:**

There were no differences in AF risk factors except for diabetes between the two groups. The average rate of Dex administration was 0.3 ± 0.2 μg/kg/h. There were also no differences between the groups in heart rate during the daytime, central venous pressure, body temperature, white blood cell count, serum level of C-reactive protein, catecholamine use, beta-blocker use, and amount of fentanyl. AF developed in one patient in the Dex group (6.3 %) and ten patients in the non-Dex group (34.5 %) during the observation period, and the difference was significant (*p* = 0.035). None of the risk factors for AF was significantly associated with AF in univariate analysis; however, multivariate logistic regression analysis using age, Dex use, and beta-blocker use, extracted because their *p* values in univariate analysis were not exceeding 0.15, showed that Dex use was the only factor associated with the development of AF (*p* = 0.045, odds ratio 9.75 [1.05–90.8]).

**Conclusions:**

The results suggest that adequate sedation with Dex during the nighttime can reduce the incidence of AF in cardiovascular surgery patients after extubation.

## Background

Atrial fibrillation (AF) is reported to occur in up to 40 % of patients in the immediate postoperative period after cardiovascular surgery despite improvements in anesthesia, surgical technique, and other medical therapies [1–4]. Patients who develop AF after cardiovascular surgery have increased mortality, morbidity, and length of hospital stay with increased health care costs [5–8].

The pathogenesis of postoperative AF is thought to be multifactorial. Preoperative factors such as increased age, reduced left ventricular function, atrial fibrosis and enlargement, and preexisting electrocardiographic abnormalities are thought to contribute to the development of AF. In addition, intraoperative factors such as reperfusion injury, systemic inflammation, hemorrhage, and hemostasis may also play a role in postoperative AF [1, 9]. The precise contribution of each of these risk factors and the role of postoperative cardiac autonomic tone in the development of AF have not been clearly defined [5, 10–13].

Dexmedetomidine (Dex), an alpha-2 agonist, has unique effects providing both sedation and analgesia by acting on central alpha-2 receptors in the locus ceruleus. These properties are useful in the intensive care unit (ICU) for relieving anxiety and pain and synchronizing mechanical ventilation with spontaneous respiratory effort [14–17]. Dex can also be used for sedation after tracheal extubation because it has little respiratory depression compared with gamma-aminobutyric acid receptor agonists, which are generally used in the ICU [14, 16, 17].

In our ICU, we previously did not administer sedative drugs to patients after tracheal extubation. However, as the use of Dex became widespread, we started to administer this agent to more patients after extubation. In our current clinical practice, we infuse Dex only during the nighttime to facilitate a natural sleep cycle. This practice helps maintain a circadian rhythm and promotes respiratory rehabilitation during the daytime.

Considering the sympathoinhibitory and anxiolytic action of Dex, there is the possibility that Dex might reduce the incidence of AF, one of the major complications after cardiovascular surgery. Therefore, we conducted this retrospective study to determine whether Dex affected the incidence of AF in cardiovascular surgery patients after extubation.

## Methods

This was a retrospective study that was approved by the ethics committee at Yamagata University Faculty of Medicine. Due to the retrospective study design under our routine clinical practice, written informed consent of an individual patient was waived.

### Sedation protocol in our ICU

In our ICU, we administered propofol and fentanyl throughout the day to patients under mechanical ventilation after cardiovascular surgery to produce Richmond Agitation Sedation Scale (RASS) between −2 and −3. The administration rate of propofol was 0.5–3 mg/kg/h and that of fentanyl was 0.05–2 μg/kg/h. We started a spontaneous breathing trial (SBT) when the patient’s body temperature, white blood cell count, and C-reactive protein levels tended to recover normally after surgery. We decided to extubate when patients met our standards for tracheal extubation: a PaO2/FIO2 ratio was >200 (at positive end-expiratory pressure of 5 cmH_2_O and pressure support of 5 cmH_2_O), PaCO_2_ was 35–50 mmHg, respiratory rate was <25 breaths per minute, and patients were also capable of deep breathing and had small amount of sputa during SBT. Just before tracheal extubation, we stopped the administration of propofol and kept infusing fentanyl only if patients felt some pain. After weaning patients from mechanical ventilation, the use of Dex as a sedative agent on the day of extubation was left to the discretion of the attending physician, intensivist, or cardiothoracic surgeon. When sedation with Dex was started, it was administered during 2 nights only from 8 p.m. to 6 a.m. following the protocol of Dex administration in our ICU. We set 2 nights after tracheal extubation as the basic administration period because of the restriction on Dex administration of 24 h at that time. Dex was adjusted to a rate of 0.2–0.7 μg/kg/h to obtain adequate sedation as assessed by RASS. If patients seemed to be delirious with incoherent behavior and distraction, we administrated haloperidol without increasing the Dex dose. Dex could be used throughout the day when patients were agitated or suffered from delirium during the daytime.

### Patients

**Fig. 1:**
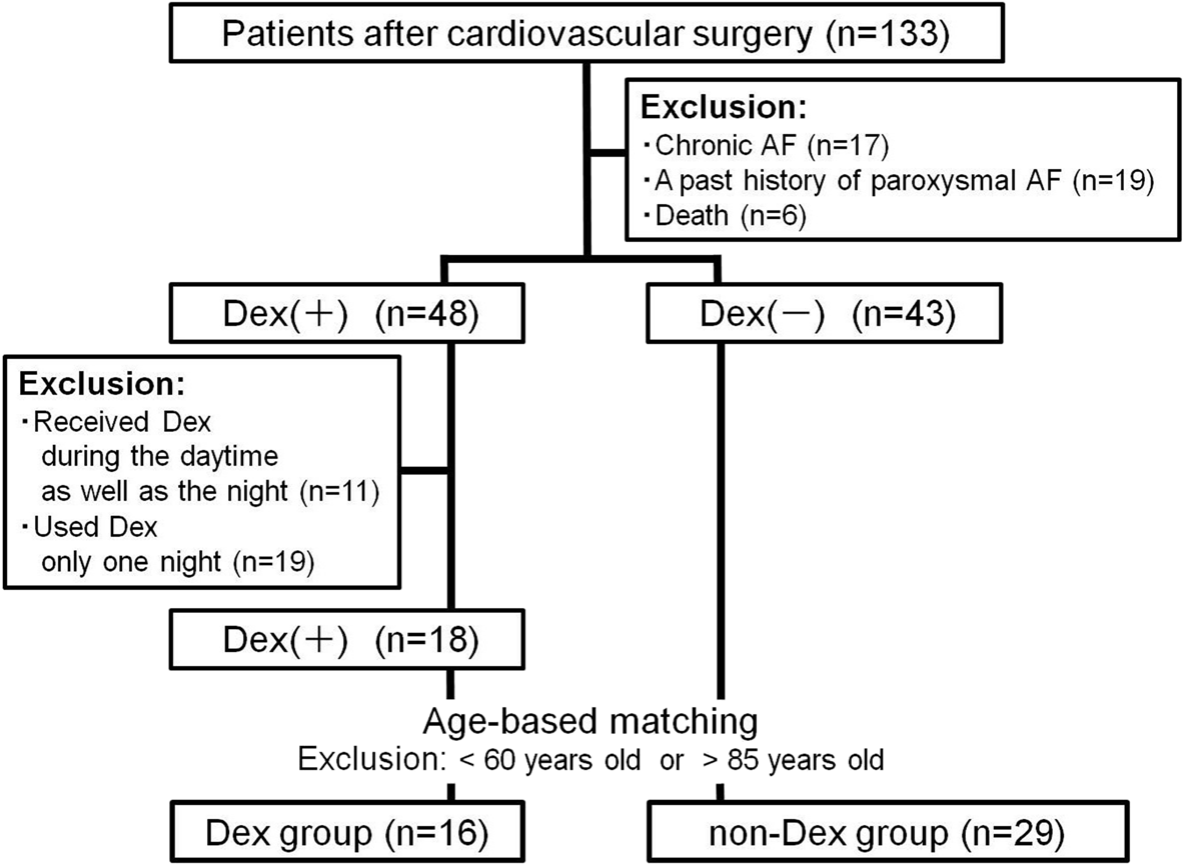
Patients enrollment flow diagram. *AF* atrial fibrillation, *Dex* dexmedetomidine

The majority of patients in our ICU had undergone cardiovascular surgery. We included a total of 133 adult patients in this study who were admitted to our ICU after cardiovascular surgery from November 2009 to November 2010. We excluded 36 patients who had chronic AF or a past history of paroxysmal AF in the preoperative or intubation period and 6 patients who died in the ICU after surgery. Of the remaining patients, 48 received Dex and 43 did not receive any sedative drugs. We excluded 30 patients who received Dex due to protocol deviation: 11 patients received all-day administration, and 19 were sedated for only 1 night. We also excluded patients under 60 years old or over 85 years old to be age-matched in both groups, because the patients over 80 years were included only in the group administered with Dex and the patients under 60 years only in the group not administered with Dex. This accounted for the exclusion of 2 patients from the 18 that received Dex and 14 patients from the 43 that did not receive Dex or any sedative drugs. The remaining patients were divided into a Dex group (*n* = 16) and a non-Dex group (*n* = 29) (Fig. 1).

### Data collection

**Fig. 2:**
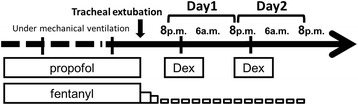
Definition of the observation period. Day 1: 24 h from 8 p.m. on the extubation day to 8 p.m. on the next day. Day 2: 24 h from 8 p.m. on the next day to 8 p.m. 2 days later following the extubation day. *Dex* dexmedetomidine

The primary outcome was the incidence of AF in cardiovascular surgery patients after tracheal extubation. We retrospectively examined the ICU records to identify the occurrence of AF during the 2-day period after tracheal extubation, because Dex was generally administered during this period at that time. We defined the observation period as follows: 24 h from 8 p.m. on the extubation day to 8 p.m. on the next day as day 1 and from 8 p.m. on the next day to 8 p.m. 2 days later following the extubation day as day 2 (Fig. 2). Our ICU staff recorded the onset of AF in the ICU chart when they detected AF on the electrocardiogram that was displayed on the bedside monitor. We diagnosed AF whether it was temporary or continuous, and the diagnosis was made without reviewing the electrocardiogram. The following demographics and risk factors for AF were also collected from the ICU chart: age, gender, body mass index (BMI), the Acute Physiology and Chronic Health Evaluation (APACHE) II score during the first 24 h of ICU stay, the Sequential Organ Failure Assessment (SOFA) score just before tracheal extubation, ICU stay, intubation day, hypertension, valvular disease, heart failure, left ventricular hypertrophy, diabetes, smoking, heart rate (HR), central venous pressure (CVP), body temperature, white blood cell count, serum level of C-reactive protein, catecholamine (dopamine and/or dobutamine) use, beta-blocker use, and fentanyl use [18]. For sequential data such as HR, CVP, and body temperature, we used the 11 a.m. records in the ICU charts as the patients’ daytime data and the 11 p.m. records as the nighttime data.

### Statistics

All statistical analyses were performed with R software (version 2.13.0). Variables were compared between groups using an unpaired *t*-test or a chi-squared test. The factors associated with AF were determined by Fisher’s exact analysis and multivariate logistic regression analysis. To control for potential confounders, a multiple logistic regression model with forward selection was used. The entry probability was *p* = 0.15 or less, and the removal probability was *p* > 0.15 in Fisher’s exact analysis [19]. A *p* value <0.05 was regarded as significant in this study.

## Results and discussion

### Results

There was no difference between the Dex group and the non-Dex group in age (71.3 vs 69.6 years.) or gender (M/F: 12/4 vs 17/12). Although the APACHE II score during the first 24 h of ICU stay was statistically higher in the Dex group (12.4 vs 10.5, *p* = 0.048), the SOFA score just before tracheal extubation was not significantly different (5.6 vs 6.1). The number of intubation days was greater in the Dex group (4.4 vs 2.3 days); however, there was no difference in the length of ICU stay (7.6 vs 6.0 days).

Diabetes, one of the risk factors for AF, was higher in the non-Dex group, while the other risk factors (BMI, hypertension, valvular disease, heart failure, ventricular hypertrophy, and smoking) were not significantly different between the two groups (Table 1). All patients were admitted to our ICU after cardiovascular surgery, such as valve replacement (31.3 % [5/16] vs 41.4 % [12/29]), coronary artery bypass graft (CABG) (25.0 % [4/16] vs 44.8 % [13/29]), valve replacement + CABG (6.3 % [1/16] vs 3.4 % [1/29]), thoracoabdominal aorta replacement (12.5 % [2/16] vs 0 % [0/29]), total arch replacement + CABG (18.8 % [3/16] vs 0 % [0/29]), and other procedures (6.3 % [1/16] vs 10.3 % [3/29]). The main operative procedures were valve replacement and CABG in both groups. More patients in the Dex group tended to undergo major surgeries such as thoracoabdominal aorta replacement or total arch replacement.

**Table 1:** Patient demographic and baseline characteristics

	Dex group (*n* = 16)	Non-Dex group (*n* = 29)	*p* value
Age (years)	71.3 ± 6.2	69.6 ± 6.7	0.4
60–69/70–79/80–85(*n*)	6/9/1	15/13/1	
Gender (M/F)	12/4	17/12	0.27
BMI (kg/m^2^)	22.3 ± 2.5	22.3 ± 3.6	0.36
APACHE II score during the first 24 h of ICU stay	12.4 ± 3.1	10.5 ± 2.9	0.048
SOFA score just before tracheal extubation	5.6 ± 1.4	6.1 ± 1.3	0.22
ICU stay (days)	7.6 ± 3.4	6.0 ± 2.1	0.06
Intubation (days)	4.2 ± 3.2	2.3 ± 1.0	0.005
Hypertension [*n*, (%)]	12 (75.0)	22 (75.9)	0.95
Valvular disease [*n*, (%)]	7 (43.8)	15 (51.7)	0.60
Heart failure [*n*, (%)]	0 (0)	3 (10.3)	0.18
Left ventricular hypertrophy [*n*, (%)]	6 (37.5)	8 (27.6)	0.49
Diabetes [*n*, (%)]	1 (6.3)	13 (44.8)	0.008
Smoking [*n*, (%)]	10 (62.5)	17 (58.6)	0.79
HR on preoperative ECG (bpm)	62.4 ± 11.1	66.3 ± 13.6	0.33

The data are presented as mean ± standard deviation or the number of patients (%)

*BMI* body mass index, *APACHE II score* Acute Physiology and Chronic Health Evaluation II score, *ICU* intensive care unit, *SOFA score* Sequential Organ Failure Assessment score, *HR* heart rate, *ECG* electrocardiogram

We examined patients’ data during the 2-day period after tracheal extubation by review of the ICU records. The average rate of Dex infusion during the nighttime in the Dex group was 0.3 ± 0.2 μg/kg/h. We did not use the hypnotic drugs during the observation period. There was statistically no difference in HR during the daytime, CVP, body temperature, serum level of C-reactive protein, catecholamine (dopamine and/or dobutamine) use, beta-blocker use, and the amount of fentanyl between the two groups. HR during the nighttime was significantly lower in the Dex group (Table 2).

**Table 2:** Patients’ data by ICU chart review in the Dex and the non-Dex groups

	Day 1			Day 2		
	Dex group (*n* = 16)	Non-Dex group (*n* = 29)	*p* value	Dex group (*n* = 16)	Non-Dex group (*n* = 27)	*p* value
Dex dose (μg/kg/h)	0.3 ± 0.1	0		0.3 ± 0.2	0	
HR daytime (bpm)	79.5 ± 7.4	84.2 ± 12.8	0.18	79.0 ± 8.7	82.8 ± 14.9	0.37
HR nighttime (bpm)	73.5 ± 13.0	84.4 ± 9.3	0.002	69.9 ± 11.3	84.3 ± 9.6	<0.001
CVP (mmHg)	6.6 ± 2.7	7.5 ± 2.7	0.36	6.9 ± 2.4	7.5 ± 3.4	0.59
Body temperature (°C)	37.5 ± 0.4	37.7 ± 0.4	0.08	37.5 ± 0.5	37.6 ± 0.4	0.27
WBC (10^3^/μl)	12.3 ± 4.8	11.8 ± 4.3	0.76	10.5 ± 4.7	10.2 ± 3.3	0.80
CRP (mg/dl)	8.8 ± 4.6	9.2 ± 5.1	0.82	7.8 ± 4.4	8.2 ± 4.0	0.76
Catecholamine use ≧3 μg/kg/m	2/16	9/29	0.17	5/16	5/27	0.27
Beta-blocker use	8/16	13/29	0.74	8/16	13/27	0.91
Amount of fentanyl (μg)	383.1 ± 339.2	429.3 ± 346.7	0.67	121.9 ± 140.6	179.3 ± 231.9	0.37

The data are presented as mean ± standard deviation or the number of patients (%). Day 1: 24 h from 8 p.m. on the extubation day to 8 p.m. on the next day. Day 2: 24 h from 8 p.m. on the next day to 8 p.m. 2 days later following the extubation day. Catecholamine: dopamine and/or dobutamine

*ICU* intensive care unit, *Dex* dexmedetomidine, *HR* heart rate, *CVP* central venous pressure, *WBC* white blood cell count, *CRP* serum level of C-reactive protein

AF occurred in one patient in the Dex group (6.3 %) and in ten patients in the non-Dex group (34.5 %) during the 2-day observation period after tracheal extubation. The lower incidence of AF in the Dex group was statistically significant (*p* = 0.035) (Table 3). In univariate analysis, none of the risk factors for AF was significantly associated with AF. Multivariate logistic regression analysis using age, Dex use, and beta-blocker use, extracted because their *p* values in univariate analysis were not exceeding 0.15, showed that Dex use was associated with the occurrence of AF (*p* = 0.045, odds ratio 9.75 [1.05–90.8]) (Table 4).

**Table 3:** Incidence of atrial fibrillation during the 2-day observation period after tracheal extubation

		Dex group	Non-Dex group	*p* value
Incidence of atrial fibrillation	Total [*n*, (%)]	1/16 (6.3)	10/29 (34.5)	0.035
	Day 1	1/16 (6.3)	6/29 (20.7)	0.20
	Day 2	0/16 (0)	7/27 (25.9)	0.05

Day 1: 24 h from 8 p.m. on the extubation day to 8 p.m. on the next day. Day 2: 24 h from 8 p.m. on the next day to 8 p.m. 2 days later following the extubation day

**Table 4:** Risk factors of atrial fibrillation

	*p* value	Odds ratio (95 %CI)
Univariate analysis		
Age	0.14	
Gender	0.72	
BMI	0.73	
Dexmedetomidine	0.067	
Catecholamine (dopamine and/or dobutamine)	0.52	
Beta-blocker	0.15	
Fentanyl	0.2	
CRP	0.52	
Hypertension	0.42	
Valvular disease	0.74	
Heart failure	1	
Left ventricular hypertrophy	1	
Diabetes	0.72	
Smoking	0.48	
APACHE II score	0.7	
SOFA score	0.33	
Intubation days	1	
Multivariate analysis		
Age	0.13	1.1 (0.97–1.24)
Dexmedetomidine	0.045	9.75 (1.05–90.8)
Beta-blocker	0.36	0.69 (0.32–1.51)

Univariate analysis was conducted by Fisher’s exact test. Multivariate logistic regression analysis was conducted using age, Dex, and beta-blocker, extracted because their *p* values in univariate analysis were not exceeding 0.15

*BMI* body mass index, *CRP* serum level of C-reactive protein, *APACHE II score* Acute Physiology and Chronic Health Evaluation II score, *SOFA score* Sequential Organ Failure Assessment score

### Discussion

Our results suggested that the incidence of AF decreased in cardiovascular surgery patients who received Dex after tracheal extubation. The incidence of AF was associated with Dex administration based on multivariate logistic regression analysis.

There are different views about the relationship between Dex and AF based on previous clinical studies. It was stated that Dex had an antiarrhythmic property during the perioperative period in patients undergoing surgery for congenital heart disease, which was produced through its action on cerebral imidazoline receptors [3]. On the other hand, it was reported that alpha-2 agonists had little effect on supraventricular tachyarrhythmia [4], and also, Dex was not associated with a significant reduction in the risk of AF in adult patients [15]. In these previous literatures compared with our study, there was a difference in that the patients received a 24-h administration of Dex during mechanical ventilation just after ICU admission. Whereas, the patients in our study received Dex only during the nighttime after tracheal extubation. We considered that this difference of administration of Dex would lead to another viewpoint on the relation between DEX and AF in our study. There were few studies focused on the relationship between Dex and AF after tracheal extubation.

AF was reported to occur in almost 50 % of patients after major cardiovascular surgeries such as total arch replacement and was higher in these patients than in those with other cardiovascular surgeries such as valve replacement and CABG [20, 21]. It was also reported that the occurrence of AF after such major surgeries peaked on day 2, and about half was widely distributed as well after day 3 [22]. In our study, the surgical stress in the Dex group was considered more severe taking into account the operative procedure and the APACHE II score during the first 24 h of ICU stay. It was considered that the intubation day was longer in the Dex group because they underwent more major surgeries than in the non-Dex group. However, the incidence of AF was significantly lower in the Dex group. This different finding compared with previous studies may have been due to the effect of Dex in addition to the fact that we collected all patient data after tracheal extubation. It was presumed that patients in both groups had a similar clinical status after extubation in our study, because there was no difference in the SOFA score just before tracheal extubation between the two groups. Furthermore, many patients in the non-Dex group that had a high incidence of AF had diabetes that has been reported to be a risk factor for AF; however, diabetes was not associated with the incidence of AF in the univariate analysis (*p* = 0.72).

Dex has unique effects to provide sedation and relieve anxiety by acting on alpha-2 receptors in the locus ceruleus and directly attenuating the sympathetic nerve in the spinal cord [15]. Dex is considered to induce a sedative response that exhibits properties similar to those of natural sleep, because it represents a similar pattern of c-Fos expression as seen during normal nonrapid eye movement sleep (a decrease in activity in the locus ceruleus and tuberomammillary nucleus and an increase in the ventrolateral preoptic nucleus) [23]. Natural sleep at night facilitates recovery from physical and mental fatigue. Therefore, endogenous catecholamine production decreases during the day as well as the night [4], and it is possible that this may account for the reduction in AF by Dex. In addition, Dex might have a direct antiarrhythmic effect to reduce the incidence of AF in our ICU patients. Cerebral nuclei that are rich in alpha-2 receptors are activated by Dex, such as the dorsal motor nucleus of the vagus nerve, the nucleus ambiguous, and the nucleus of the tractus solitarius. The enhanced vagal activity affects myocyte cyclic adenosine monophosphate and the calcium ion current and consequently leads to prolongation of repolarization and the effective refractory period. This evidence supports a hypothesis for an antiarrhythmic action of Dex [24].

An important limitation of our study is that it is a retrospective study. The reasons are unclear why physicians in our ICU decided to use Dex to provide sedation after tracheal extubation. It was just left to the discretion of the attending physician at that time and was never intentionally made. Furthermore, it is possible that we did not accurately diagnose AF because we evaluated the presence of AF based only on the ICU chart, and we cannot review the electrocardiogram at the time of AF occurrence. There is also a limitation that the patients have been examined at different periods after surgery in the Dex group (4.2 ± 3.2 days) and the non-Dex group (2.3 ± 1.0 days). It has been reported that a peak of postoperative AF was day 2 focused on CABG and valve surgery, and more than 95 % of AF episodes occurred before day 5 [2]. While it has been reported that the distribution of onset of AF after major surgeries such as thoracoabdominal aorta replacement and total arch replacement showed a peak on day 2, but also about half was widely distributed as well after day 3 [22]. In the DEX group in our study, 31.2 % patients underwent such major surgeries, so AF possibly occurred at the observation period. Therefore, we consider that it is worth comparing the occurrence of AF at the different periods between two groups.

## Conclusions

In our study, the incidence of AF after tracheal extubation decreased significantly in cardiovascular surgery patients that received Dex only during the nighttime. It is probably because Dex has unique effects to reduce sympathetic tone and endogenous catecholamine production as well as to promote adequate sedation like natural sleep. Because this study is a small retrospective study, there are some limitations on the differences on the observation period, the operative procedure, and the patient characteristics such as diabetes between the Dex group and the non-Dex group. A prospective investigation making a distribution of the background of the study is needed in a larger population to confirm the effect of Dex on AF reduction.

## Abbreviations

AF: atrial fibrillation
Dex: dexmedetomidine
ICU: intensive care unit
RASS: Richmond Agitation Sedation Scale
SBT: spontaneous breathing trial
BMI: body mass index
APACHE: Acute Physiology and Chronic Health Evaluation
SOFA: Sequential Organ Failure Assessment
HR: heart rate
CVP: central venous pressure
CABG: coronary artery bypass graft
